# Ball Tip Technique for S2AI Screw Placement in Sacropelvic Fixation: A Comparative Study with Conventional Freehand Technique

**DOI:** 10.1111/os.13196

**Published:** 2022-01-02

**Authors:** Zhenhai Zhou, Cheng Tu, Honggui Yu, Jiachao Xiong, Zhiming Liu, Shengbiao Ma, Wenqiang Deng, Kai Cao

**Affiliations:** ^1^ The Orthopaedic Hospital The First Affiliated Hospital of Nanchang University Nanchang China; ^2^ Lushan Convalescent Center and Clinic of People's Liberation Army of China Jiujiang China

**Keywords:** Ball tip technique, Complications, Pelvic fixation, S2 alar‐iliac screws, Sacropelvic fixation

## Abstract

**Objective:**

To evaluate the efficiency of the ball tip technique for S2AI screw placement and introduce this technique.

**Methods:**

Sixty‐three patients who underwent pelvic fixation with S2AI screws were retrospectively reviewed. They were 29 males and 34 females with an average age of 59.6 ± 12.5 years. Among these patients, 35 patients (14 males and 21 females with an average age of 58.8 ± 11.3 years) received ball tip technique and 28 patients (15 males and 13 females with an average age of 63.7 ± 12.6 years) received conventional freehand technique. Ball tip technique was used in ball tip technique group. After a pedicle probe just penetrated the sacroiliac joint, a ball‐tipped probe consisting of a ball shaped metal tip with a flexible shaft was malleted to make a guide track within ilium. This ball‐tipped probe could bend automatically away from the cortex and forward through the cancellous bone when the tip met the cortical lamina of ilium, which can avoid penetration. After repeating the procedures, a guide hole was gradually formed. S2AI screw was inserted along the guide hole after tapping. In the conventional freehand group, S2AI screw was placed according to the conventional method. Postoperative computed tomography (CT) was used to assess the accuracy of screws. The time cost of screw insertion and screw‐related complications were recorded. Independent t‐test was used to compare the time cost between ball tip group and conventional freehand group. A chi‐square test was used to compare the accuracies of the ball tip group with the conventional group.

**Results:**

There were 35 patients (70 S2AI screws) in ball tip group and 28 patients (56 S2AI screws) in conventional freehand group. No screw‐related complication occurred in all patients. Time costs were 9.8 ± 4.5 mins in ball tip group and 20.2.0 + 8.6 mins in conventional freehand group, respectively (*P* < 0.05). Four screws penetrated iliac cortex in the ball tip group *vs* 10 screws in conventional freehand group (5.7% *vs* 17.9%) (*P* < 0.05).

**Conclusions:**

The ball tip technique enhances the accuracy of screw placement and has less time cost compared with conventional freehand technique.

## Graphical abstract

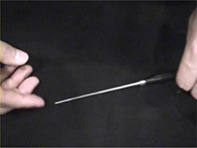

This work introduced a novel technique named ball tip technique for S2AI screws placement. Ball tip technique enhances the accuracy of S2AI screw placement.

## Introduction

To achieve solid osseous fusion across the lumbosacral junction has historically been, and continues to be, a challenge in spine deformity surgery involved in sacropelvic unit^1^. Multiple techniques of pelvic fixation were introduced, including Galveston rods, transiliac bars, iliac bolts, and iliosacral screws^2^. These techniques frequently require separate incisions for placement or the use of offset connectors, result in more surgical time cost and morbidity^3^. The S2‐alar‐iliac (S2AI) technique was initially used for the pediatric and adult deformity patients described by Sponseller and Kebaish^4^, ^5^, and S2AI has the advantages of having a low profile in line with proximal screw, avoiding extra incision and better biomechanics, which contributes to less screw‐related complications^6^, ^7^. Considering the above advantages, in recent years, S2AI techniques have become the most commonly used methods for sacropelvic fixation and the freehand technique has obtained popularity. However, free hand technique of S2AI is technically demanding in some cases, particular for less experienced surgeons. To increase the accuracy and safety of the S2AI technique, in a previous study, the authors developed the computer‐assisted 3D template guided technique (TGT) to place S2AI and the results were satisfactory^8^. After being implemented in some cases, problems still arose from TGT, in that the accuracy of TGT was crucially influenced by the printing materials and the precision of printing. If the printed guide template did not closely match the dorsal surface of the sacrum, this technique would bring about significant deviation, which compelled the surgeons to give up the template technique during the surgery in a few cases. Therefore, in recent years, a more reliable free‐hand technique, named ball tip technique, has been developed instead of the conventional freehand technique for S2AI screws placement. Ball tip probe consists of a ball‐shaped metal tip with a metal semi‐flexible shaft. Ball tip technique was initially introduced in thoracic spinal pedicle screw insertion, as described by Kota Watanabe^9^. Inspired by this method, the authors developed a special probe with characteristics of the semi‐flexibility of metal shaft. This special ball tipped probe could seek the cancellous channel between medial and lateral iliac cortices automatically when gently tapped on the tail with a mallet. The aims of this study are: (i) to introduce a novel technique named ball tip technique for S2AI screw placement; (ii) to evaluate the efficacy of ball tip technique for S2AI screws; (iii) to compare the accuracy and safety of ball tip technique with conventional freehand technique for S2AI screw placement.

## Methods

This is a retrospective study, and it was approved by Ethic Committee of The First Affiliated Hospital of Nanchang University.

### Patients Data

Sixty‐three consecutive patients who underwent spinopelvic fixation using S2AI screws by a senior surgeon over 40‐month period (April 2015–August 2018) were reviewed retrospectively. The inclusion criteria: adult patients underwent sacropelvic fixation with S2AI. The exclusion criteria: S2AI screws were inserted with other techniques such as 3D template guided technique (TGT). The patients comprised 29 males and 34 females with an average age of 59.6 ± 12.5 years. Among these patients, 35 patients (14 males and 21 females with an average age of 58.8 ± 11.3 years) received ball tip technique (a total of 70 S2AI screws) and 28 patients (15 males and 13 females with an average age of 63.7 ± 12.6 years) received conventional freehand technique (a total of 56 S2AI screws) (Table 1). After surgery, pelvic CT scan was used to assess the position of screws in both groups and any screw‐related complications were recorded. Time cost of screw placement were also recorded and compared between two groups.

**TABLE 1:** General information of the patients

Characteristics	Freehand group	Ball tip group	Value of t or *X* ^2^	*P* value
No. of Cases	n = 28	n = 35	*X* ^*2*^ = 1.153	*P* = 0.318
	Male = 15	Male = 14		
	Female = 13	Female = 21		
Mean age (y)	63.7 ± 12.6	58.8 ± 11.3	t = 1.625	*P* = 0.109
Weight (kg)	76.7 ± 7.5	72.5 ± 9.3	t = 1.937	*P* = 0.057
Height (cm)	170.3 ± 5.9	166.5 ± 10.3	t = 1.736	*P* = 0.088
BMI (kg/m^2^)	26.4 ± 1.8	26.1 ± 2.1	t = 0.565	*P* = 0.574
Diagnosis			—	—
Adult Spinal deformity	20	26		
Lumbosacral infection	3	4		
Lumbosacral TB	5	4		
Lumbosacral tumor	0	1		

### Surgical Procedure

#### Anesthesia and Exposure

Under the general anesthesia, the patient was positioned prone on the operation table. All the patients underwent posterior approach and the lumbosacropelvic posterior elements were meticulously exposed.

#### Define the Starting Point of S2AI Screw

The starting points for S2AI screw placement were located lateral to the midpoint between the S1 and S2 foramina, and in line with the proximal S1 and L5 anchors. In ball tip group, the cortical bone of the sacral dorsal lamina at the appropriate starting points was removed using high‐speed drill.

#### Probe the Optimal Trajectory of S2AI Screw

**Fig. 1:**
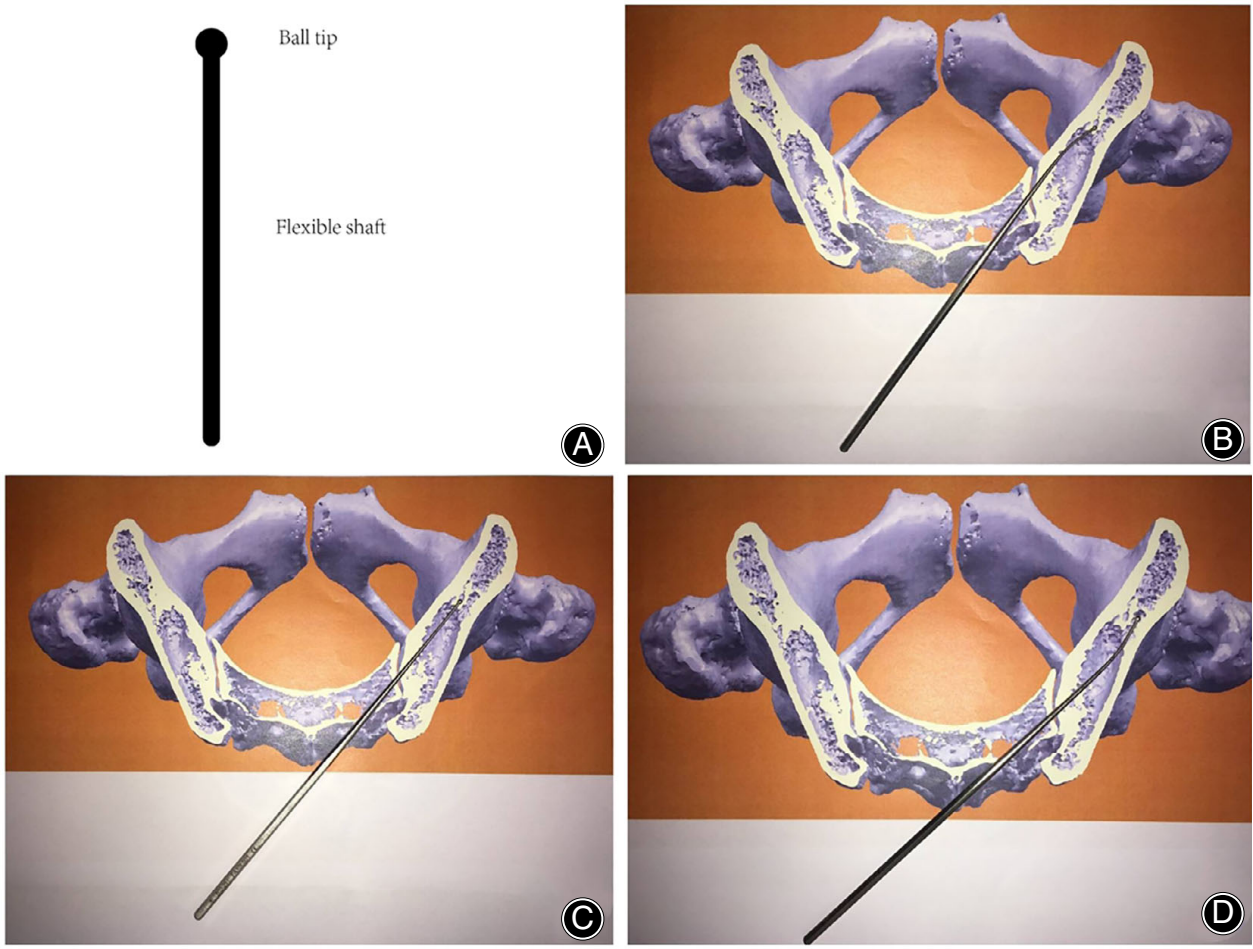
Construction of a ball tip probe and the sketch map. (A) A ball tip probe consists of a ball shaped metal tip with a metal flexible shaft. It is used to create a guide track. (B–D) The behavior of a ball tip probe in the ilium. The probe will bend automatically when it meets the cortical lamina of ilium, which can make a guide track in ilium ultimately.

A 2‐mm blunt‐tipped pedicle probe is advanced toward the sacroiliac (SI) joint through S2 alar by aiming the point two‐finger width proximal to the ipsilateral greater trochanter. When the pedicle probe penetrated the SI joint and just broke through the iliac joint surface, a special ball‐tipped probe was used instead of pedicle probe and inserted along the primary track hole. The probe was then gently malleted to advance it through the cancellous channel to make a guiding track in ilium. The ball tip probe consisted of a ball‐shaped metal tip with a semi‐flexible metal shaft. It had the characteristics of metal's semi‐flexibility and could bend automatically when meeting the lateral or medial cortical lamina of ilium, which could make a track hole within ilium ultimately (Fig. 1). After repeating the ball tip probe in‐and‐out procedure several times, a soft feeler was used to palpate the integrity of the created track hole. After that, a Lenke pedicle probe was used to widen the track hole. In the conventional freehand group, the starting point is the same as that mentioned above. The following procedure is the same as the method described by Kebaish and Sponseller previously^5^.

#### Screw Insertion

**Fig. 2:**
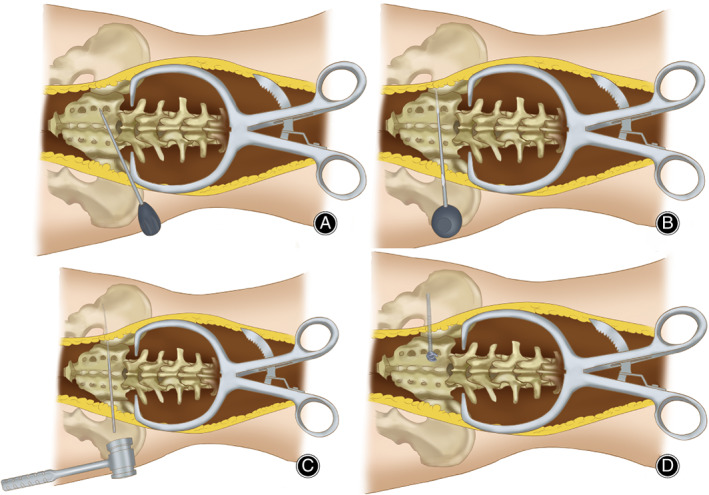
A sketch map of ball tip technique. Figure 2 shows the procedure of ball tip technique. The starting points for S2AI screw placement are located lateral to the midpoint between the S1 and S2 foramina, and in line with the proximal S1 and L5 anchors. A 2‐mm blunt‐tipped pedicle probe is advanced toward the sacroiliac (SI) joint through S2 alar by aiming the point two‐finger width proximal to the ipsilateral greater trochanter (A). When the pedicle probe penetrated the SI joint and just broke through the iliac joint surface, ball‐tipped probe was used instead of pedicle probe and inserted along the primary track hole, then gently malleted to advance through the cancellous channel to make a guiding track in ilium. (B and C). S2AI screw was inserted through the track hole after surrounding and tapping (D).

When the trajectory was confirmed, the S2AI screw was inserted through the track hole after surrounding and tapping (Fig. 2). In both groups, all the screws were placed by the same senior spine surgeon.

### Outcome Measures

#### Postoperative Accuracy Evaluation of Screw Placement

**Fig. 3:**
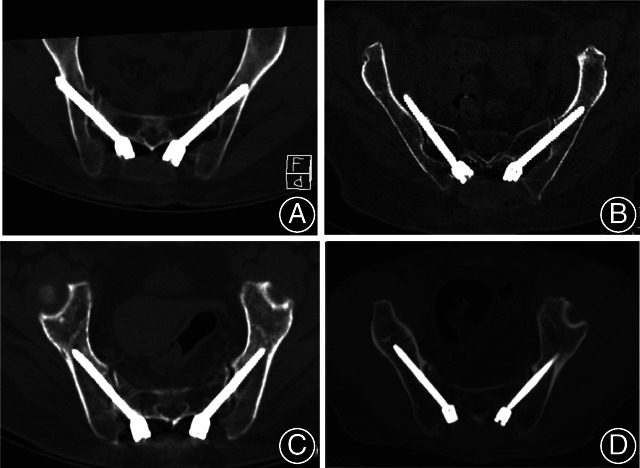
The positions of S2AI screws placed by two techniques. (A) Showed inaccurate screw insertion by conventional freehand technique and the screw penetrated the iliac cortex posteriorly. (B) Showed inaccurate screw insertion by conventional freehand technique and the screw penetrated the iliac cortex anteriorly. (C and D) Showed excellent screws position by ball tip technique.

Fine‐cut (width of 1 mm) CT is used to assess the accuracy of screw placement after surgery. The screw contained in the iliac cancellous bone was defined as being accurately placed. By contrast, screw that violated or broke medial or lateral cortex, or the cortex of sciatic notch, was defined as inaccurate position (Fig. 3). The screws' breach levels were divided into four grades according to the previous description by Oh^10^. Grade 0 was defined as no breach and the screw was located within the iliac cancellous bone completely; grade 1 was defined as mild breach as the breach distance was <3 mm out of the adjacent cortex; grade 2 was defined as moderate breach as the breach distance was 3–6 mm out of the adjacent cortex; grade 3 was defined as severe breach as the breach distance was >6 mm out of the adjacent cortex. Mild, moderate, and severe violations are considered as inaccurate screw placement.

#### Time Cost Assessment of Screw Placement

Time cost of unilateral screw placement was recorded and compared between the two groups. Timing started at the time of finding the starting point and finished at the time the screw was inserted completely without a revision.

#### Evaluation of Safety of Screw Placement

Any cortex violation‐related and screw‐related complication was recorded and compared between two groups to evaluate safety of screw placement, including major vessel injury, nerve injury, screw broken, and screw loosened. The complications occurred in follow‐up were considered to be no relation with accuracy of screws and were not evaluated.

### Statistical Analysis

Statistical analysis was performed using SPSS 17.0 (SPSS Inc., Chicago, IL). Data are presented as the mean ± standard deviation. Independent t‐test was used to compare the time cost between ball tip group and conventional freehand group. A chi‐square test was used to compare the accuracies of the ball tip group with the conventional free hand group. Statistical significance was defined as *P* < 0.05.

## Results

### The Evaluation of Accuracy of the Screws in Both Groups

A total of 126 screws were inserted in 63 patients. In ball tip group, a total of four screws (in four patients) were placed with mild cortical breaches and no screw was placed with moderate to severe cortical breaches. No breach occurred anterior, while four were posterior. In conventional freehand group, a total of 10 screw (in nine patients) breaches were noted, including four screws were placed with mild cortical breaches and six screws were placed with moderate to severe cortical breaches. Two breaches occurred anterior, while eight were posterior. A significant difference in the accuracy of screws placement was observed between two groups (5.7% *vs* 17.9%) (*X* ^*2*^ *=* 4.645, *P* < 0.05) (Table 2).

**TABLE 2:** Comparison of accuracy of S2AI screws insertion in ball tip and conventional freehand group

Characteristics	Freehand group	Ball tip group	Value of t or *X* ^2^	P value
Total screws	56	70	—	—
No breach	46	66	*X* ^*2*^ *=* 4.645	*P* = 0.03
Breaches	10	4		
Breach grade			—	—
0 (0 mm)	46	66		
1 (<3 mm)	4	4		
2 (3–6 mm)	4	0		
3 (>6 mm)	2	0		
Breach direction			—	—
No breach	46	66		
Anterior	2	0		
Posterior	8	4		
Inferior	0	0		

### Comparison of Time Cost of Two Techniques

Time cost of unilateral S2AI screw placement were 9.8 ± 4.5 mins in ball tip group and 20.2 + 8.6 mins in conventional freehand group, respectively. There is a significant difference in the time cost between two groups. Ball tip technique for S2AI screw placement has less time cost compared with conventional freehand technique (t = −8.738, *P* < 0.01).

### Complications

No cortex violation‐related or screw‐related complications occurred in any of the patients.

## Discussion

### Ball Tip Technique Enhances Accuracy of S2AI Screw Placement

Cortex violation of the ilium would occur when S2AI screws were placed by free hand technique. The cortical breach of an S2AI screw may result not only in decrease fixation strength but also potentially injury of the major vessels, particularly the internal iliac artery with an anterior breach and the superior gluteal artery with a caudal breach^7^. O'Brien and colleagues reported a 15% rate of posterolateral cortex violation in 10 cadaveric specimens^11^. Shillingford and colleagues found an overall 7% rate of posterior cortical perforation and a 1% rate of anterior cortex violation^12^. Posterolateral cortex violation did not endanger any major neurovascular structures. Although anterior pelvic violation and inferior screw violation were rare, they can result in severe complications and devastating consequences. Anterior violations can result in injuries of important visceral or neurovascular structures located anterior to the pelvis^13^. Moreover, inferior screw violation into the sciatic notch may result in injuries of sciatic nerve, pudendal nerves, or internal pudendal vessels as they pass over and under the piriformis muscle within the notch^14^. In recent years, several techniques were used for S2AI screw placement, including navigation, freehand technique, and TGT. However, freehand technique obtained popularity because of convenience. Some limitations of navigation and TGT, including expense and complexity, limited them being widely use. In this study, to further enhance the accuracy or S2AI screw placement by freehand technique, the authors adopted ball tip technique for S2AI screw placement. Ball tip technique applied in thoracic pedicle screw insertion for adolescent idiopathic scoliosis patients was initially described by Kota Watanabe^9^. They found that ball tip technique could enhance the accuracy of pedicle screw placement in clinical and cadaveric study. Whether the ball tip technique could be feasibly used for S2AI screw insertion and increase the accuracy of S2AI screw placement has not been, to our knowledge, studied previously in the available literature. The results of our study demonstrated an overall 5% rate of cortical perforation in ball tip group, which significantly increases the accuracy compared with the result of 17.9% rate of cortical perforation in conventional freehand group. However, neither of the two different techniques gave rise to major neurologic or vascular complications.

**Fig. 4:**
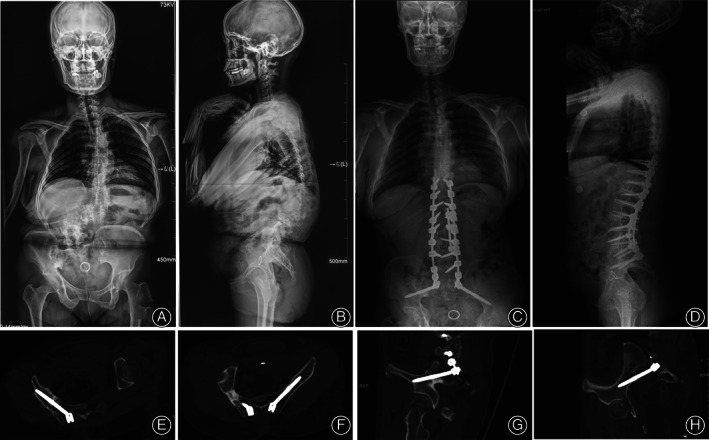
Typical case 1 managed by ball tip technique. A 44‐year‐old female presented severe congenital thoracolumbar deformity with a morphological abnormality of right sacroiliac joint, in which the hypertrophy is obvious under the overload stress. Also, the pelvis is asymmetrical with the right side being anteriorly rotational (A, B). The anomaly made the S2AI screw insertion by conventional free hand technique to be difficult. Surgeon conducted the ball tip technique to solve this problem perfectly. Postoperative X‐rays showed the satisfactory correction and good sagittal and coronal realignments with a sacropelvic fixation (C, D). Postoperative CT scan indicated the perfect positions of S2AI screws bilaterally in the axial and sagittal plates of screws without any cortex breach (E–H).

**Fig. 5:**
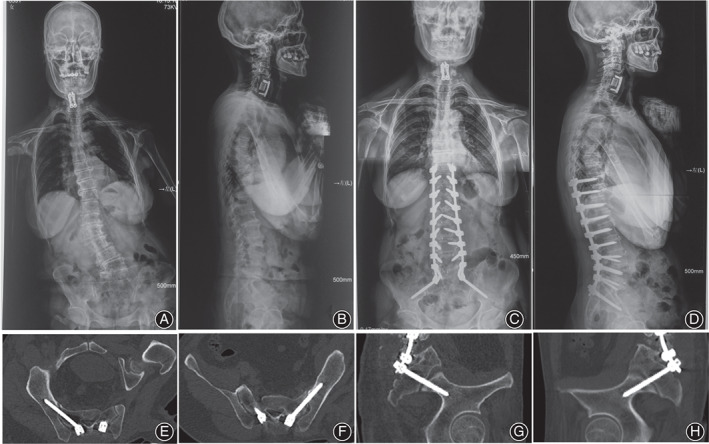
Typical case 2 managed by ball tip technique. A 60‐year‐old female presented trunk shift. She underwent an anterior cervical discectomy and fixation (ACDF) at local hospital 3 years ago. Preoperative X‐rays showed a coronal imbalance. (A, B) Surgeon adopted the ball tip technique for S2AI screw placement. Postoperative X‐rays showed the satisfactory correction and good sagittal and coronal realignments with a sacropelvic fixation (C, D). Postoperative CT scan indicated the perfect positions of S2AI screws bilaterally in the axial and sagittal plates of screws without any cortex breach (E–H).

**Fig. 6:**
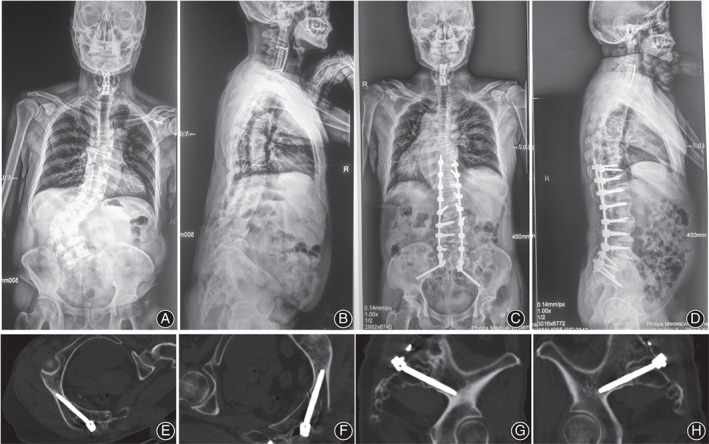
Typical case 3 managed by ball tip technique. A 68‐year‐old male presented severe back pain and weakness of low limbs. Rigid lumbar scoliosis. He underwent an anterior cervical discectomy and fixation (ACDF) at local hospital 5 years ago. Preoperative X‐rays showed a rigid lumbar scoliosis and asymmetric pelvis (A–D). Surgeon adopted the ball tip technique for S2AI screw placement. Postoperative X‐rays showed the satisfactory correction and good sagittal and coronal realignments with a sacropelvic fixation (Fig. 3C, D). Postoperative CT scan indicated the perfect positions of S2AI screws bilaterally in the axial and sagittal plates of screws without any cortex breach (E–H).

Furthermore, compared with conventional freehand technique, ball tip technique decreased the frequency of using C‐arm, which would significantly reduce radiation exposure to the patient and surgeon. In the conventional freehand technique, the surgeon had to find the teardrop and ensured the guided probe and the inserted screw inside the teardrop under repeated intraoperative fluoroscopy. On the contrary, in procedure of ball tip technique, the surgeon took the special probe with characteristics of semi‐flexible metal shaft to find the track hole in the cancellous channel automatically, rather than having to continue watching the probe inside the teardrop, which obviously reduces the frequency of using C‐arm. Consequently, the efficacy of S2AI screw insertion was significantly improved. Especially for some patients with severe lumbosacral deformity and rotational pelvis, ball tip technique significantly decreased frequency of intraoperative fluoroscopy and simplified surgical procedure (Figs 4, 5, 6).

Some surgeons who used conventional freehand technique reported that only 5% of the S2AI screws demonstrated moderate to severe cortical breaches, all of which perforated the pelvis posteriorly and all only involved the distal part of the screw tip depending on the horizontal and caudal angles^12^; but in an anther study, the rate of cortex penetration was high, reaching 15%^11^. In this study, stricter evaluation criteria were adopted in which mild to severe cortex breaches were considered as inaccurate insertion. In ball tip technique group, the total rate of penetration was 5.7% (4/70) but there was no moderate to severe cortex breach. In conventional freehand group, although the total rate of penetration was 17.9%, the rate of moderate to severe cortex breaches was 10.7% (6/56). Our result further demonstrated that ball tip technique enhanced the accuracy of S2AI screw placement, especially avoiding moderate to severe penetration.

### Ball Tip Technique Reduces Time Cost for S2AI Screw Placement

In this study, the time cost between ball tip technique and conventional freehand technique were also compared, our result demonstrated that ball tip technique could statistically decrease the time cost of screw placement from 20.2 ± 8.6 min to 9.8 ± 4.5 min. Less surgical time means less intraoperative blood loss and lower rate of wound infection. Less operation time may also decrease surgeon fatigue, improve concentration, and potentially correlate with positive patient outcomes^15^.

### Limitations of Current Study

There were limitations to this study. The ball tip technique was not generalized in pediatric patients, whether this technique could be used in pediatric patients needs to be further investigated. This study is a small series in a single center and the patients were not randomized for both techniques in this retrospective study. Limited results were obtained from this study and future studies may benefit from randomizing patients divided into ball tip *vs* conventional freehand technique. Additionally, although our result demonstrated that the ball technique is useful and reliable for S2AI screw placement in this study, it still needs to be emphasized that it may not be helpful to a surgeon who has no experience in placing S2AI screws, since a mistake in the identification of starting point could lead to missed screw placement even if the ball tip technique is used.

### Conclusion

Ball tip technique for S2AI screw placement is not only a reliable but also a practical technique. This technique significantly enhances the accuracy of S2AI screw placement and improves the efficacy by reducing the time cost for S2AI screw placement in spine surgery involved in sacropelvic unit compared with conventional freehand technique.

## Ethics Approval and Consent to Participate

This study was approved by the Ethics Committee of The First Affiliated Hospital of Nanchang University.

## Consent for Publication

Written informed consent was acquired from each patient to authorize treatment, imageology findings, and photographic documentation. The patients consented to the publication of their pictures as well as their anonymous and clustered data.
